# Grouper RIP2 inhibits Singapore grouper iridovirus infection by modulating ASC-caspase-1 interaction

**DOI:** 10.3389/fimmu.2023.1185907

**Published:** 2023-05-08

**Authors:** Xin Zhang, Siting Wu, Zetian Liu, Hong Chen, Jiaming Liao, Jingguang Wei, Qiwei Qin

**Affiliations:** ^1^ Guangdong Laboratory for Lingnan Modern Agriculture, College of Marine Sciences, South China Agricultural University, Guangzhou, China; ^2^ Department of Biological Sciences, National University of Singapore, Singapore, Singapore; ^3^ Laboratory for Marine Biology and Biotechnology, Pilot National Laboratory for Marine Science and Technology (Qingdao), Qingdao, China

**Keywords:** *Epinephelus coioides*, receptor interacting protein 2, Singapore grouper iridovirus, ASC, caspase-1

## Abstract

**Introduction:**

Receptor interacting protein 2 (RIP2), serves as a vital sensor of cell stress, is able to respond to cell survival or inflammation, and is involved in antiviral pathways. However, studies on the property of RIP2 in viral infections in fish have not been reported.

**Methods:**

In this paper, we cloned and characterized RIP2 homolog from orange-spotted grouper (Epinephelus coioides) (EcRIP2) and further discussed the relevance of EcRIP2 to EcASC, comparing the influences of EcRIP2 and EcASC on the modulation of inflammatory factors and the NF-κB activation to reveal the mechanism of EcRIP2 in fish DNA virus infection.

**Results:**

Encoded a 602 amino acid protein, EcRIP2 contained two structural domains: S-TKc and CARD. Subcellular localization signified that EcRIP2 existed in cytoplasmic filaments and dot aggregation patterns. After SGIV infection, the EcRIP2 filaments aggregated into larger clusters near the nucleus. The infection of SGIV could notably up-regulate the transcription level of the EcRIP2 gene compared with lipopolysaccharide (LPS) and red grouper nerve necrosis virus (RGNNV). Overexpression of EcRIP2 impeded SGIV replication. The elevated expression levels of inflammatory cytokines induced by SGIV were remarkably hindered by EcRIP2 treatment in a concentration-dependent manner. In contrast, EcASC treatment could up-regulate SGIV-induced cytokine expression in the presence of EcCaspase-1. Enhancing amounts of EcRIP2 could overcome the down regulatory effect of EcASC on NF-κB. Nevertheless, increasing doses of EcASC failed to restrain the NF-κB activation in the existence of EcRIP2. Subsequently, it was validated by a co-immunoprecipitation assay that EcRIP2 dose-dependently competed with EcASC binding to EcCaspase-1. With increasing time to SGIV infection, EcCaspase-1 gradually combined with more EcRIP2 than EcASC.

**Discussion:**

Collectively, this paper highlighted that EcRIP2 may impede SGIV-induced hyperinflammation by competing with EcASC for binding EcCaspase-1, thereby suppressing viral replication of SGIV. Our work supplies novel viewpoints into the modulatory mechanism of RIP2-associated pathway and offers a novel view of RIP2-mediated fish diseases.

## Introduction

Receptor-interacting protein (RIP) kinases are vital molecules in the modulation of cell death and inflammation processes. The family of RIP kinases shares a homologous serine-threonine kinase domain. RIP1 and RIP3 are the most investigated members of the RIP kinase family, which mediate cell death processes (1) . RIP2 lacks the RIP homotypic interaction motif (RHIM) found in the cell death associated RIP1 and RIP3, containing an N-terminal kinase domain and a C-terminal caspase-recruitment domain (CARD) (2). The CARD of RIP2 makes it possible for its binding with other proteins containing CARD, such as caspase-1 (3–5). This binding results in the induction of the RIP2-regulated signal cascade, thereby contributing to the NF-κB activation (6). NF-κB is a group of transcription factors that functions in immune regulation, cell apoptosis, proliferation, as well as survival (7). Multiple receptors activate NF-κB *via* diverse signaling pathways in response to bacterial products, cytokines, and cellular stress conditions.

Caspase-1, as a cysteine protease, plays vital and distinctive roles in the innate immune reaction through attracting diverse CARD-CARD interaction partners. The CARD procaspase-1 participates in inflammasome formation through type I interactions with ASC (Apoptosis-associated Speck-like protein containing a CARD domain), while RIP2-regulated activation of NF-κB involves type III interactions (8). The inflammasome refers to supramolecular signaling complexes that assemble in response to microbial stimuli. It signals the activation of caspases and mature proinflammatory cytokines and is implicated in innate immune defense (9). In the meantime, ASC is able to enhance intercellular communication and IL-1β maturation, and also serves as a dual mediator to advance or restrain the NF-κB activation based on the impacting parameters of the stimulation (10). In mammals, ASC and RIP2 appear to play opposite roles in the activation of NF-κB and inflammatory pathways (5). Our previous study confirmed that ASC from orange-spotted grouper (*EcASC*) could interact with Caspase-1 and up-regulated the expression levels of proinflammatory parameters. Besides, *EcASC* blocked the activation of NF-κB (11).

However, high levels of cytokines can also harm the host. The immune system may be activated to the limit or out of control, even creating a “cytokine storm”. Viruses like SARS-CoV and MERS-CoV can contribute to extreme immune attacks in the body, thereby leading to severe respiratory disease and even death (12). Lianhuaqingwen was reported to exert its drug activity against the novel coronavirus (SARS-CoV-2) by suppressing viral replication and decreasing the cytokine secretion from host cells (13). Currently, it has been demonstrated that RIPK2 mediates protection against seasonal influenza A virus (IAV)-induced morbidity and mortality by negatively regulating IL-18 and IFN-γ production in lung interstitial cells and immune cells. This is vital for the protection from IAV-induced immunopathology during IAV infection (14). In contrast, in bony fish, only reports have evaluated the participation of RIP2 in the antimicrobial and pro-inflammatory reactions of goldfish macrophages (15). It has also been elucidated that mixed plasticizers exacerbate hepatocyte apoptosis in grass carp *via* the NOD2-RIP2-NF-κB pathway (16), but studies on the property of RIP2 in viral infections in fish have not been reported. The grouper, also known as *Epinephelus* spp., is a significant mariculture fish species in Southern Asia and China. Nevertheless, the recent continual eruptions of viral diseases have contributed to serious pecuniary losses to the grouper industry. Singapore grouper iridovirus (SGIV), as a large cytoplasmic DNA virus, is the grouper and seabass’s primary pathogen (17, 18). Infection with SGIV in diseased fish can result in enlargement with hemorrhage of spleen and multifocal degeneration of splenic degeneration, thus causing high mortality rates in groupers (19, 20). Multiple immune genes have been cloned by concentrating on the antiviral immune network to excavate the protection and therapy of grouper virus diseases (21). A homologue of RIP2 (*EcRIP2*) was discovered in orange-spotted grouper (*Epinephelus coioides*). In our work, the antiviral capability of EcRIP2 in the process of SGIV infection was investigated and its relevance to key proteins in the signaling pathway was assessed. It is of interest to discuss whether the binding of caspase-1 to ASC directly competed with the binding to RIP2, which results in the NF-κB activation, thus contributing to cell survival and preventing the virus-induced excessive inflammation. Our results will offer novel viewpoints into the property of fish RIP2 in virus infection.

## Materials and methods

### Fish, cells, and viruses

Juvenile orange groupers, weighting 30-40 g, were purchased from Maoming Marine Fish Farm (Guangdong, China). These fishes were placed in a recirculating seawater system of laboratory under the condition of 24-28°C and reared two times per day for two weeks. Then three groupers were randomly selected to detect whether the fish was infected with bacteria or viruses. Twelve tissues were extracted from 6 healthy fish, immediately frozen in liquid nitrogen, and stored at -80°C.

Grouper spleen (GS) cells were constructed in our laboratory and are currently kept in our laboratory. GS cells were cultivated in Leibovitz’s L15 medium containing 10% FBS (Gibco, USA) at 28°C (22). The SGIV’s virus stocks were propagated in GS cells, and the vital titer was measured in GS cells (23). Virus stocks were retained at -80°C.

### EcRIP2 cloning and bioinformatics prediction

The primers (**Table 1**) were designed following the expressed sequence tag (EST) sequences of EcRIP2 from the grouper spleen transcriptome (24), and the PCR amplification was utilized for cloning the full-length EcRIP2 sequence. Afterward, the BLAST program (http://www.ncbi.nlm.nih.gov/blast) was implemented for analyzing the sequence of EcRIP1, and the Conserved Domains program (https://www.ncbi.nlm.nih.gov/cdd/) was employed for forecasting the conserved domains or motifs. Next, the performance of amino acid alignments was achieved with Clustal X1.83 software, and edited by the GeneDoc program. In MEGA 6.0 software, the conduction of the phylogenetic analysis was completed with the neighbor-joining (NJ) method.

**Table 1:** Primers utilized in this research.

Primers	Sequences (5’-3’)
EcRIP2-ORF-F	ATGATAAATCCTCAGAGACTTCCC
EcRIP2-ORF-R	CTACTGCTGCACTCTGCTCCTGGTG
EcRIP2-RT-F	GGCTTCCATCGTCGTTT
EcRIP2-RT-R	GGCTTGAGGGTGAGGTTAT
EcRIP2-GFP-F	GCCTCGAGCTATGATAAATCCTCAGAGACTTCCC
EcRIP2-GFP-R	CGAGAATTCGACTGCTGCACTCTGCTCCTGGTGATAC
EcRIP2-HA-F	GCGAATTCATGATAAATCCTCAGAGACTTCCC
EcRIP2-HA-R	CGCTCGAGCTACTGCTGCACTCTGCTCCTGGTG
EcASC-HA-F	ATGCCCCCAAAAACCAAAAGAAAGG
EcASC-HA-R	TCACTTCTTCCCCATAAGCTCATCG
EcCaspase-1-GFP-F	CGCAA GCT TCGATGGCAGACGAGCTCGCCAG
EcCaspase-1-GFP-R	GCGGATCCTTAGAGGCCTGGGAAGAAGTAG
EcCaspase-1-HA-F	ATGGCAGACGAGCTCGCCAGAGTG
EcCaspase-1-HA-R	TTAGAGGCCTGGGAAGAAGTAGAAG
Actin-RT-F	TACGAGCTGCCTGACGGACA
Actin-RT-R	GGCTGTGATCTCCTTCTGCA
SGIV MCP-F	GCACGCTTCTCTCACCTTCA
SGIV MCP-R	AACGGCAACGGGAGCACTA
SGIV ICP-18-F	ATCGGATCTACGTGGTTGG
SGIV ICP-18-R	CCGTCGTCGGTGTCTATTC
SGIV VP19-RT-F	TCCAAGGGAGAAACTGTAAG
SGIV VP19-RT-R	GGGGTAAGCGTGAAGAC
EcIL-1β-RT-F	AACCTCATCATCGCCACACA
EcIL-1β-RT-R	AGTTGCCTCACAACCGAACAC
Primers	Sequences (5’-3’)
EcIL-6-RT-F	GGTTGGTCCAAGGTGTGCTTA
EcIL-6-RT-R	CTGGGATTGTCGAGGTCCTT
EcIL-8-RT-F	GCCGTCAGTGAAGGGAGTCTAG
EcIL-8-RT-R	ATCGCAGTGGGAGTTTGCA
EcTNF-α-F	GTGTCCTGCTGTTTGCTTGGTA
EcTNF-α-R	CAGTGTCCGACTTGATTAGTGCTT

### RNA isolation and quantitative real-time-PCR

SV Total RNA Isolation Kit (Promega, USA) was utilized for the extraction of the total RNAs. The total RNA quality was estimated with 1% agarose gel electrophoresis. The synthesis of the first-strand cDNAs was completed by reverse transcription of the total RNAs with the application of the ReverTra Ace kit (Toyobo, Japan).

The transcriptional expression levels of virus and host immune genes were evaluated with qRT-PCR in an Applied Biosystems Quant Studio 3 Real-Time Detection System (Thermofisher, USA). Every experiment was replicated in 3 times. The primers for genes are exhibited in **Table 1**. The target gene levels were calculated with the 2^−ΔΔCT^ method, with β-actin as a loading control. The data were depicted as mean ± standard deviation.

### RIP2 expression patterns in grouper

The samples of the head kidney, skin, heart, brain, liver, intestine, spleen, kidney, stomach, gill, as well as blood that had been harvested from six healthy orange groupers were utilized for RNA extraction and subsequent qRT-PCR for addressing the tissue distribution of EcRIP2. GS cells were respectively subjected to infection with PBS, lipopolysaccharide (LPS), SGIV, as well as RGNNV, to screen for EcRIP2-sensitive exogenous stimuli. Cell samples were obtained at 0, 12, 24, 36, and 48 h to examine the EcRIP2 expression level by qRT-PCR. Animal studies were ratified by the Animal Ethics Committee of the South China Agricultural University.

### Plasmid construction and cell transfection

The full-length EcRIP2 was inserted into the pEGFP-C1 vector for evaluating subcellular localization. The subsequent protein function and co-immunoprecipitation assays were employed by inserting the full-length EcRIP2 or EcASC in to pcDNA3.1-3HA vector. DNA sequencing was employed for the confirmation of all recombinant plasmids. Cell transfection was implemented with Lipofectamine 2000 transfection reagent (Invitrogen) (25). In short, GS cells, at 60-70% confluence, were seeded in 24-well or 6-well cell culture plates. After that, cells were subjected to 6-h cultivation with the plasmids and Lipofectamine 2000 mixture before replacing with a fresh normal medium.

### Microscopy observation

GS cells were transfected with the plasmids of pEGFP-C1 and pEGFP-EcRIP2. Next, cells, at 24 h after transfection, were subjected to fixation with 4% paraformaldehyde and dying with DAPI. Finally, the fluorescence was visualized with a confocal laser scanning microscope.

### Virus infection assay

For detecting how EcRIP2 and EcASC affected viral infection, cells overexpressing pcDNA3.1-3HA, pcDNA3.1-EcRIP2, or pcDNA3.1-EcASC were subjected to incubation of SGIV. The parallel cell samples were acquired for extracting RNA and synthesizing cDNA. After that, the transcription levels of the SGIV VP19 (viral protein 19), infected cell protein 18 (ICP-18), and major capsid protein (MCP) genes were estimated by qRT-PCR. We conducted each assay three times. In the meantime, the influence of elevated EcRIP2 on MCP expression was tested by western blotting.

### Dual-luciferase reporter assays

GS cells were transiently transfected the luciferase plasmids (NF-κB-Luc) coupled with the corresponding expression vectors with Lipofectamine 2000 reagent. SV40 (50 ng) was supplemented for normalizing the luciferase activities. Afterward, the harvested cells were utilized for evaluating the luciferase activities with a Dual-Luciferase^®^ Reporter Assay System kit.

### Co-immunoprecipitation assays and western blotting

Cells in a 10 × 10 cm cell culture dish underwent two days of transfection using 16 mg of DNA plasmid. The GS cells upon transfection were lysed in radio immunoprecipitation assay buffer. A Dynabeads Protein G Immunoprecipitation Kit (Invitrogen) was served for processing and analyzing the interaction of cell samples. Next, 12% SDS-PAGE was adopted for the separation of proteins. We then transferred the separated proteins onto the Immobilon-P polyvinylidene difluoride membranes. Blots were subjected to incubation with certain primary antibodies, anti-3HA or anti-MCP, at a 1:1,000 dilution, and subsequent incubation of anti-rabbit or anti-mouse IgG antibody conjugated by HRP at 1:5,000 dilution. An improved HRP-DAB chromogenic substrate kit was utilized for detecting the immunoreactive proteins.

### Data analysis

GraphPad Prism 6.0 software was adopted for all statistical analysis. *p < 0.05 or **p < 0.01 represented a statistical significance.

## Results

### Sequence characterization and tissue distribution of EcRIP2

**Figure 1:**
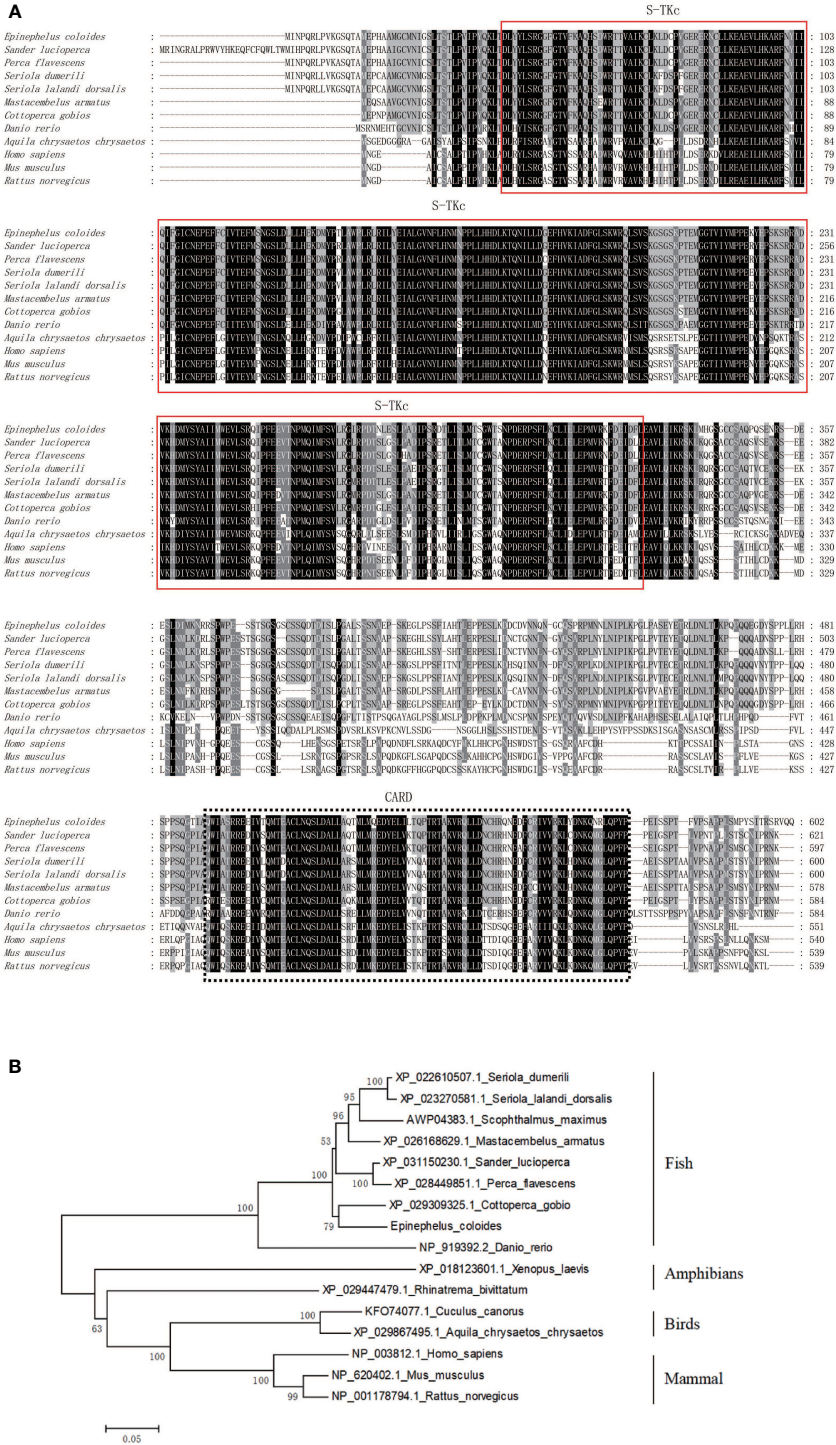
Bioinformatic analysis of EcRIP2. **(A)** Multiple sequence alignments regarding RIP2. **(B)** Phylogenetic tree constructed following amino acid sequence alignment.

The grouper transcriptome’s EST sequences were considered for amplifying the full-length cDNA of EcRIP2, which was found to contain two structural domains: S-TKc and CARD. Based on the BLAST analysis, the homology between EcRIP2 and the RIP2 of *Epinephelus lanceolatus* (XP_033507423.1) reached 99.83%. ClustalX was utilized for conducting the multiple sequence alignments and the NJ method was implemented for building a phylogenetic tree with 1,000 bootstrap replicates. Clustering of EcRIP2 was found in the Osteichthyes branch, in which the RIP2 subfamily was conserved (**Figures 1A, B**).

### Cellular localization of EcRIP2

**Figure 2:**
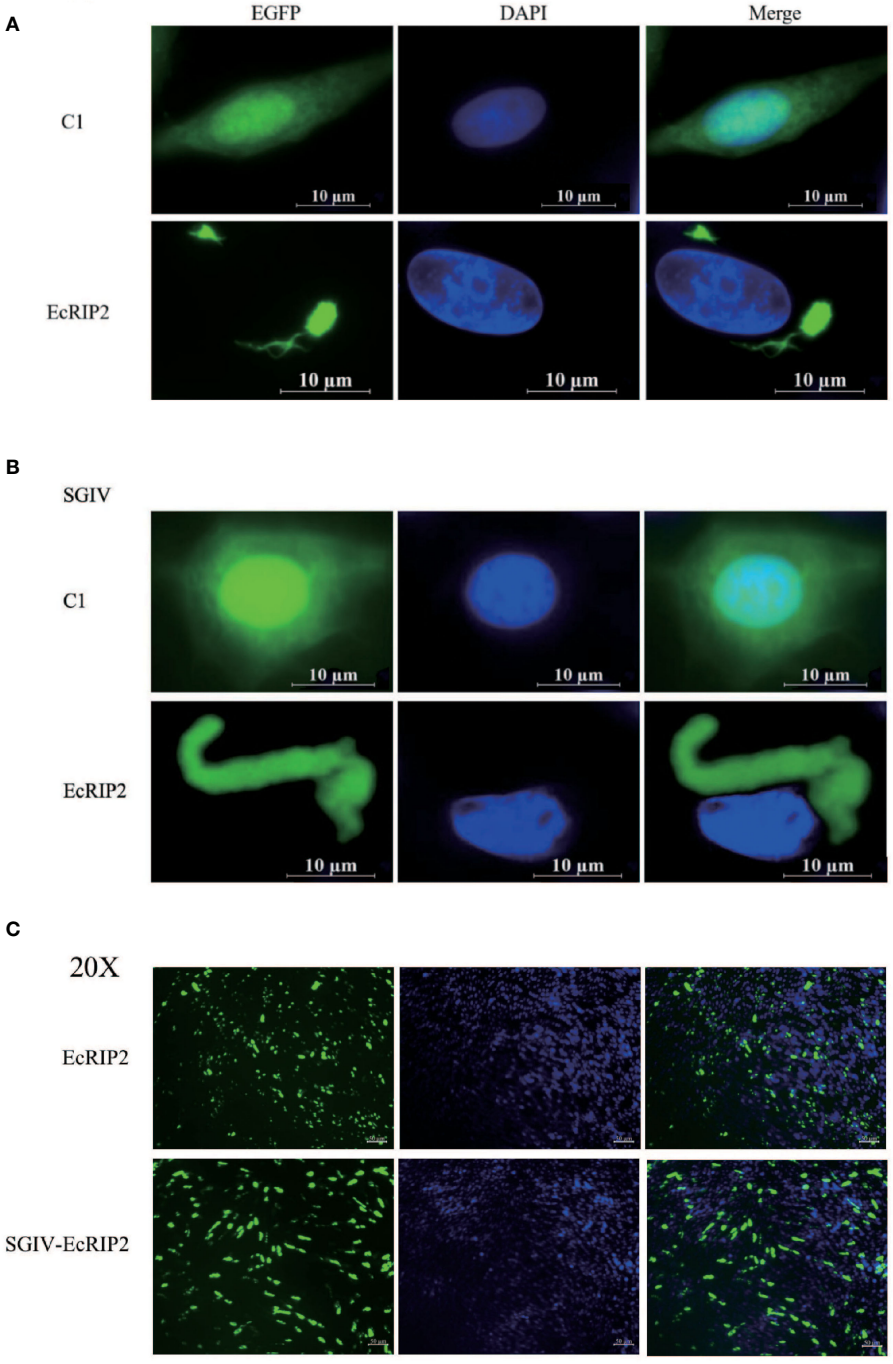
Cellular localization of EcRIP2. **(A)** Fluorescent localization of empty vector and EcRIP2 in GS cells without SGIV infection. **(B)** GS cells were transfected with pEGFP-C1 and pEGFP-EcRIP2 plasmids. Cells were infected with SGIV at 24 h post-transfection, and stained with DAPI at 12 h post-infection, and imaged under a confocal laser scanning microscopy. **(C)** The smaller images taken at low magnification (20X).

The EcRIP2 subcellular location was assessed in GS cells and SGIV-infected cells to elucidate the function of EcRIP2. The fluorescent signal of pEGFP-C1 existed in the whole cells (**Figures 2A, B**). EcRIP2 showed cytoplasmic filament and dot aggregation patterns without SGIV infection in contrast to the control group (**Figure 2A**). Upon SGIV infection, the EcRIP2 distribution formed a larger aggregation form close to the nucleus (**Figure 2B**). The smaller images taken at low magnification (40X) have been added in **Figure 2C** to allow observation of cell transfection efficiency.

### Tissue distribution and expression profiles of EcRIP2 *in vivo*

**Figure 3:**
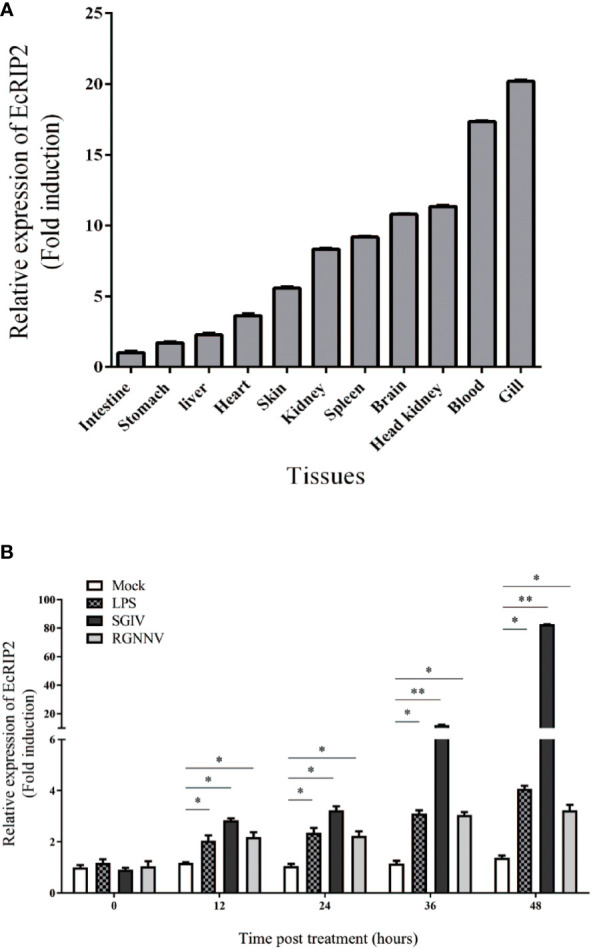
EcRIP2 expression patterns. **(A)** Tissue distribution exhibited by EcRIP2 in healthy groupers. **(B)** The temporal expression profile of EcRIP2 in GS cells under different exogenous stimuli including LPS, RGNNV, and SGIV infection. *p < 0.05; **p < 0.01.

The EcRIP2 transcript levels in various tissues from healthy juveniles were tested by qRT-PCR. EcRIP2 was found to exsit in all 11 tissues, and its high level was mainly observed in gill, blood, head kidney, brain, and spleen (**Figure 3A**). Subsequently, the temporal expression profile of EcRIP2 in GS cells under diverse exogenous stimuli was determined by qRT-PCR for characterizing the expression pattern of EcRIP2 during pathogen infection. The findings disclosed that compared with LPS and red grouper nerve necrosis virus (RGNNV), SGIV infection could remarkably up-regulate EcRIP2 transcription level (**Figure 3B**), suggesting that EcRIP2 is involved in the regulation of SGIV infection.

### Differential influences of EcRIP2 and EcASC on SGIV infection *in vitro*

**Figure 4:**
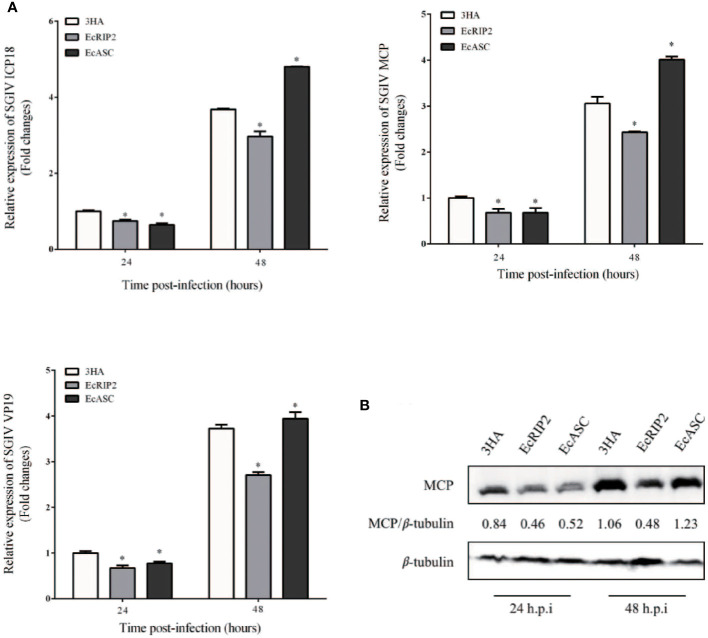
Differential effects of EcRIP2 and EcASC on SGIV infection *in vitro*. **(A)** Viral gene transcription (VGT) levels in GS cells under the transfection of pcDNA3.1-3 × HA, HA-EcRIP2, or HA-EcASC. Transfected GS cells were subjected to infection of SGIV at a multiplicity of infection (MOI) of 2 and were then acquired at 24 and 48 h post-infection (h.p.i) for confirming ICP18, MCP, and VP19 levels by qPCR. **(B)** SGIV-MCP levels in GS cells at 24 and 48 h.p.i were appraised by western blotting (n = 3, mean ± SD). *p < 0.05.

Our previous studies have demonstrated that grouper ASC interacts with caspase-1 and promotes the expression of ATP-induced inflammatory factors. However, the effect of ASC on SGIV infection is not known. Therefore, we examined the effects of grouper ASC and RIP2 on SGIV replication (11). GS cells were subjected to transfection with pcDNA3.1-EcRIP2 and pcDNA3.1-ASC, and subsequent 24- and 48-h infection with SGIV to clarify the property of EcRIP2 and EcASC in SGIV replication. EcRIP2 elevation distinctly diminished the SGIV genes (ICP18, MCP, and VP19) transcriptional levels (**Figure 4A**). When measuring the SGIV MCP protein levels at 24 h and 48 h with the same treatment, EcRIP2 restrained the MCP protein levels (**Figure 4B**). However, the elevation of EcASC advanced the transcription of ICP18, MCP, and VP19 and MCP protein level in the late period of SGIV infection (48 h) (**Figures 4A, B**).

### Differential regulation of inflammatory factors by EcRIP2 and EcASC

**Figure 5:**
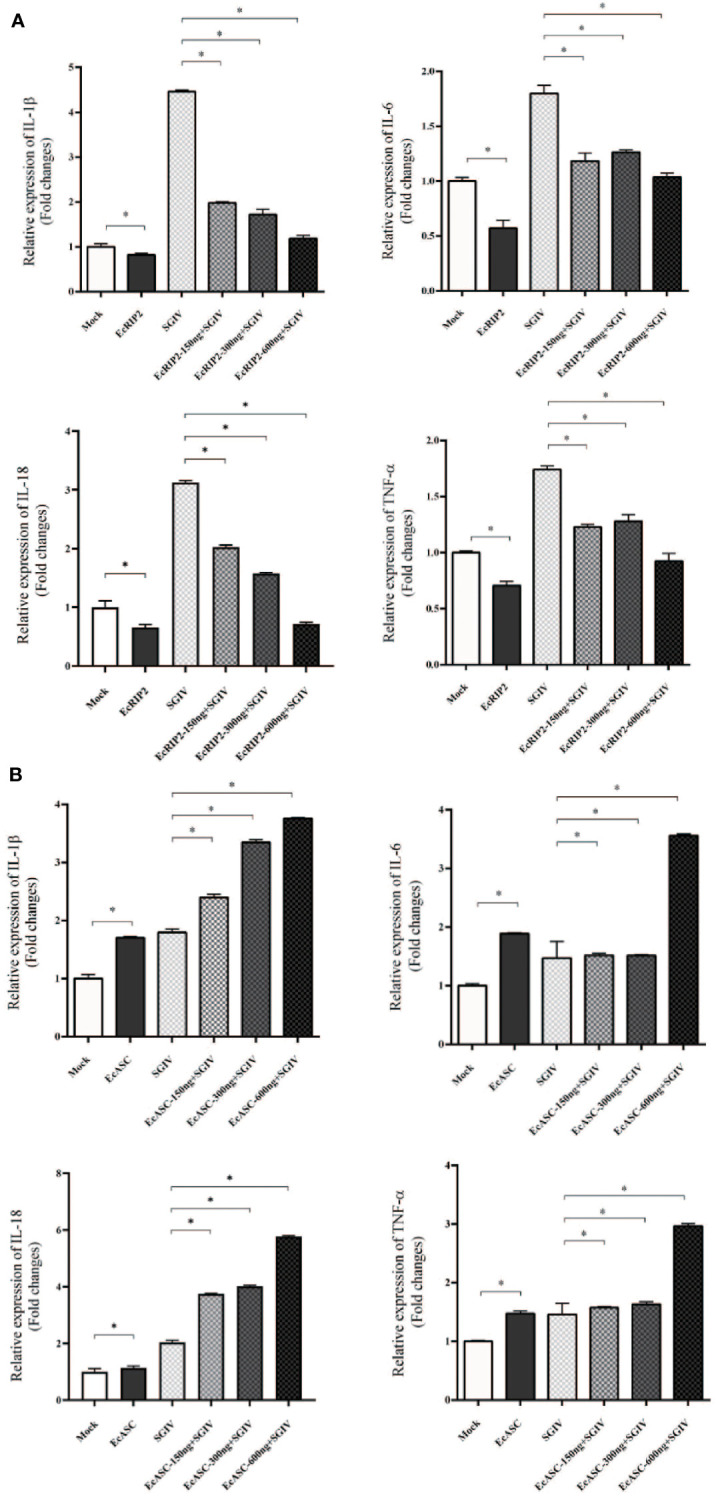
Effects of **(A)** EcRIP2 or **(B)** EcASC overexpression in the increased doses on the levels of inflammatory mediators in SGIV-infected GS cells. Data are depicted as the mean ± SD obtained from 3 independent assays. *p < 0.05, compared with mock-treated cells.

Following, the capabilities of EcRIP2 and EcASC on the host inflammation responses in the presence of caspase-1 were tested by qRT-PCR for uncovering the mechanism implicated in the properties of EcRIP2 and EcASC in SGIV infections. IL-1β, TNF-α, IL-6, as well as IL-18 mRNA levels were compared between the EcRIP2- or EcASC-overexpressed and mock-treated GS cells. We observed that EcRIP2 treatment led to reduced levels of these four cytokines (**Figure 5A**). In contrast, EcASC up-regulated SGIV-induced cytokine levels (**Figure 5B**).

### EcRIP2 up-regulates EcASC-regulated NF-κB activation in GS cells

**Figure 6:**
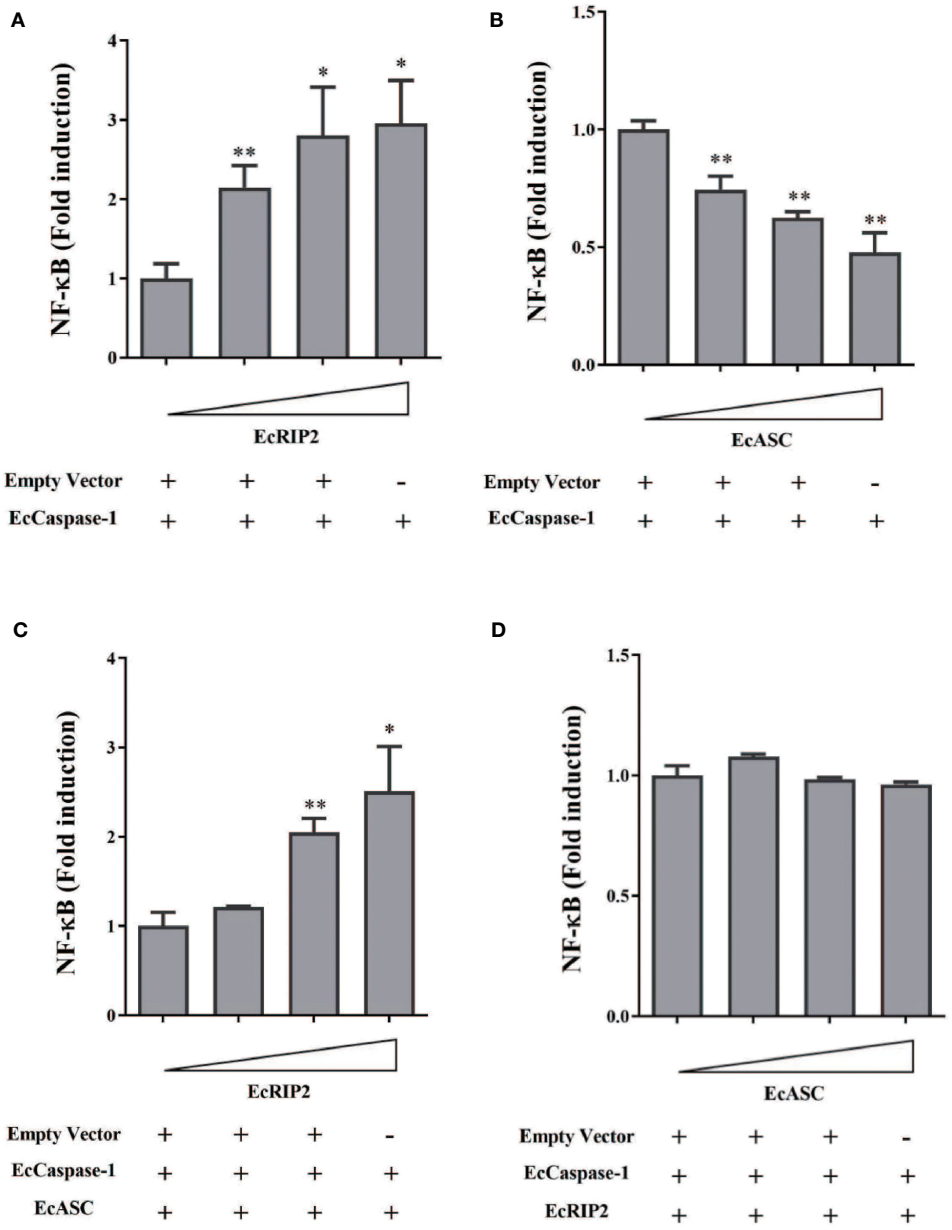
EcRIP2 up-regulates EcASC-mediated NF-κB activation in GS cells. GS cells were subjected to transfection with elevated doses of **(A)** EcRIP2 or **(B)** EcASC (0, 150, 300, and 600 ng) along with EcCaspase-1 (150 ng). **(C)** GS cells were transfected with increasing EcRIP2 (0, 150, 300, and 600 ng) along with EcASC (150 ng) and EcCaspase-1 (150 ng). **(D)** GS cells were subjected to transfection with elevated EcASC (0, 150, 300, and 600 ng) along with EcRIP2 (150 ng) and EcCaspase-1 (150 ng) (total DNA remained throughout the experiments using an empty vector). NF-κB activation was estimated by the luciferase activity assay. *p < 0.05; **p < 0.01.

Although there is a reported active role of RIP2 on the NF-κB pathway, its exact mechanism remains unsolved. The influence of EcRIP2 on the NF-κB activation in GS cells was appraised with the luciferase reporter assay system for unveiling the unique role of EcRIP2. It was observed that EcRIP2 induced NF-κB activation in the presence of EcCaspase-1 (**Figure 6A**). Previous publications have confirmed that EcASC and EcCaspase-1 could jointly block the NF-κB activation, and we have again verified that EcASC dose-dependently hindered the NF-κB activation (**Figure 6B**). Next, GS cells were subjected to transfection with EcASC and EcCaspase-1 together with elevated doses of EcRIP2 to delineate the impact of EcRIP2 on this EcASC-inhibited NF-κB activation. Total plasmid DNA was maintained throughout the experiments. Upregulation of EcRIP2 in the presence of EcASC could lead to elevated NF-κB expression, implying that the increase in the EcRIP2 amounts could counteract the EcASC-induced down-regulation of NF-κB (**Figure 6C**). Nevertheless, increasing doses of EcASC failed to inhibit the NF-κB activation in the existence of EcRIP2 (**Figure 6D**).

### EcRIP2 modifies EcASC/caspase-1 interaction

**Figure 7:**
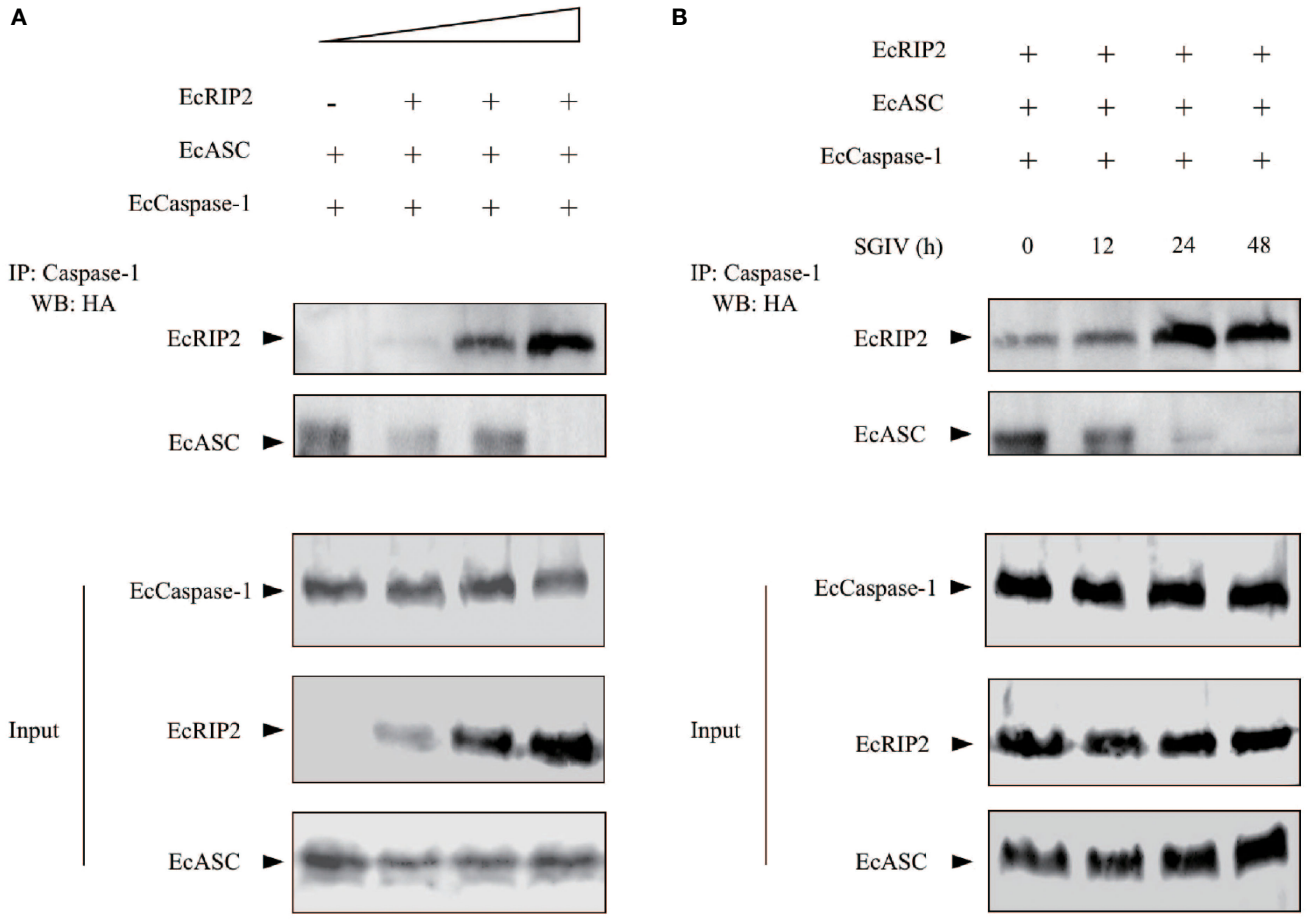
EcRIP2 affects EcASC-procaspase-1 interaction. **(A)** GS cells were subjected to transient transfection with elevating doses of EcRIP2 (0, 150, 300, and 600 ng), EcASC (150 ng), and EcCaspase-1 (150 ng). Total DNA levels remained unchanged during the transfection with an empty vector. The interaction between EcRIP2/EcASC and EcCaspase-1 was analyzed *via* the immunoprecipitation of caspase-1 by using a capturing Ab, relative to a control Ab. **(B)** Cells overexpressing EcRIP2 (300 ng), EcASC (300 ng), and EcCaspase-1 (300 ng) were infected with SGIV, and the ability of EcRIP2 or EcASC to bind with EcCaspase-1 was measured by immunoprecipitation at the time point of incremental infection. Whole cell lysates (Input) transfected with GFP-Caspase-1 and HA-RIP2 or HA-ASC were subjected to immunoprecipitation (IP) and immunoblotting (IB) with indicated antibodies.

Our previous work demonstrated that EcASC may interact with EcCaspase-1 (11). Meanwhile, in view of the functional differences between EcRIP2 and EcASC, we wanted to explore whether there were differences in a combination with EcCaspase-1. GS cells received the co-transfection of EcCaspase-1 and EcASC, in the existence or the absence of EcRIP2. The cell lysates were subjected to co-immunoprecipitation with a caspase-1 antibody. We found that EcRIP2 competed with EcASC for binding to EcCaspase-1 in a dose-dependent way (**Figure 7A**). In the meantime, the cells overexpressing EcRIP2, EcASC, and EcCaspase-1 were treated with SGIV infection. With increasing time to SGIV infection, EcCaspase-1 gradually combined with more EcRIP2 than EcASC (**Figure 7B**).

## Discussion

Numerous publications have unveiled that the members of the RIP serine/threonine kinase family are pivotal sensors of cell stress (26, 27). Initially, RIP2 was thought of as a serine-threonine kinase relates to caspase-1 (3), which was later reclassified to be a dual-specificity kinase capable of phosphorylating tyrosine residues. RIP2 can be used by the intracellular peptidoglycan sensors to participate in the NF-κB pathway to guide the innate immune reactions against bacterial and viral infections (28). In addition, ASC has also been reported to be a CARD-containing molecule that plays an important role in the induction of apoptosis and caspase-1 activation. In mammals, ASC and RIP2 play opposite roles in the activation of NF-κB (5). Our previous studies have demonstrated that grouper ASC interacts with caspase-1 and promotes the expression of ATP-induced inflammatory factors. However, the effect of ASC on SGIV infection is not known. How RIP2 functions as a vital mediator of signaling pathways and shows its antiviral property have seldom been discussed in lower vertebrates. Therefore, in this paper, we cloned and characterized EcRIP2 to unveil the influences of EcRIP2 on the modulation of inflammatory factors and the NF-κB activation, and further discussed the relevance of EcRIP2 to EcASC to reveal the mechanism of EcRIP2 in fish DNA virus infection.

EcRIP2 shares 99.83% homology with *Epinephelus lanceolatus* (XP_033507423.1). Besides, the BLAST analysis uncovered that EcRIP2 not only shares a homologous S-TKc domain with family members, but also carries a CARD that allows recruitment to other proteins containing CARD, such as caspase-1. RIP2 forms high molecular weight cytosolic complexes upon infection with intracellular/invasive bacteria, and the complexes are called RIPosomes. RIP2 is able to polymerize into long filamentous structures, which serves as signaling platform to the downstream of NOD1/2. RIP2 filament formation is necessary for NOD2-dependent NF-κB signaling (29, 30). In our paper, subcellular localization analysis signified that EcRIP2 was exhibited in cytoplasmic filament and dot aggregation patterns without SGIV infection. Upon SGIV infection, the EcRIP2 filament formed a larger aggregation form close to the nucleus. These results implied that EcRIP2 may be implicated in the induction of NF-κB activation and in the regulation of SGIV infection.

Studies on RIP2 have focused on its mediation of inflammatory reactions to bacterial infections (through the NOD1/2-signaling) (31–33), or involvement in response to certain RNA virus infections (12, 14, 34), and DNA damage (27, 35). However, in this study, the infection of SGIV that mainly causes a high death rate of grouper could distinctly up-regulate the EcRIP2 transcription level in comparison to LPS and RGNNV. Furthermore, the upregulation of EcRIP2 was determined to suppress the transcription levels of SGIV genes, consisting of ICP18, MCP, and VP19. Besides, EcRIP2 also restrained the protein content of SGIV MCP. These findings confirmed that EcRIP2 was capable of hindering the SGIV replication *in vitro*. On the contrary, the CARD-contained EcASC advanced the transcription of viral genes and MCP protein level in the late period of SGIV infection (48 h). As reported, EcASC could interact with EcCaspase-1 (11). The two proteins that both contain the CARD domain play different roles in SGIV replication, indicating that they may have functional differences.

RIP2 is critical for protection against seasonal IAV-induced immunopathology during IAV infection. Also, it was confirmed that RIP2 deficient mice (RIP2^−/−^) produced excess IL-18 *in vivo*, driving the majority of the immunopathology (14). In addition, in our paper, EcASC has been demonstrated to notably upregulate the mRNA transcript levels of inflammatory factors in the presence of EcCaspase-1. We, therefore, proposed to address the impact of RIP2 on the SGIV infection-induced immune response. The results elucidated that the elevated levels of inflammatory factors such as IL-1β, IL-6, IL-18 and TNF-α induced by SGIV were impeded by EcRIP2 treatment in a concentration-dependent way. In contrast, EcASC up-regulated SGIV-induced cytokine expression in the presence of EcCaspase-1. We speculate that excess EcASC is capable of enhancing the process of SGIV inflammatory response and accelerating cell death. In the late stage of SGIV infection, this excessive inflammatory response promotes viral release. In contrast, overexpression of EcRIP2 may partly limit the excessive immunity-induced viral proliferation.

RIP2 is considered an inducer of the NF-κB signaling. Severely reduced NF-κB activation has been observed in RIP2-deficient mice (4, 33). In this work, we intended to expound on the capability of EcRIP2 in the NF-κB signaling. The findings unveiled that EcRIP2 dose-dependently enhanced the NF-κB activation in the presence of EcCaspase-1. Previous articles have confirmed that EcASC and EcCaspase-1 can jointly inhibit the NF-κB activation (11), and we have again verified that EcASC dose-dependently hinders the NF-κB activation. Subsequently, GS cells were subjected to transfection with EcASC and EcCaspase-1 together with elevated doses of EcRIP2. Upregulation of EcRIP2 in the presence of EcASC could result in elevated NF-κB expression, demonstrating that the increase in the EcRIP2 amount could counteract the EcASC-induced down-regulation of NF-κB. Nevertheless, increasing doses of EcASC failed to inhibit the NF-κB activation in the existence of EcRIP2. We hypothesize that the dual function of EcRIP2 in mediating NF-κB might rely on the CARD’s stoichiometry among the binding proteins. The low ratios of EcASC to EcRIP2 and EcCaspase-1 may advance the link between EcRIP2 and EcCaspase-1, thereby advancing the subsequent NF-κB activation.

Other literature has indicated that caspase-1 binds to RIP2 through its CARD (4, 8, 36). Furthermore, our report has already disclosed that EcASC results in the EcCaspase-1 activation *via* its CARD interactions (11). Therefore, we speculate that EcRIP2 competes with EcASC for binding to EcCaspase-1. In subsequent research, a co-immunoprecipitation assay confirmed that EcRIP2 competed with EcASC for the binding to EcCaspase-1 in a dose-dependent way. Moreover, the cells overexpressing EcRIP2, EcASC, and EcCaspase-1 were infected with SGIV. With increasing time to the infection, EcCaspase-1 gradually combined with more EcRIP2 than EcASC. Obviously, our study highlights that EcRIP2 plays a pivotal role in communications between the signalosome and the inflammasome. We speculate that EcRIP2 inhibits SGIV-induced hyperinflammation by competing with EcASC for binding to EcCaspase-1, thereby suppressing viral replication of SGIV. These functions of EcRIP2 may be significant in controlling the SGIV-induced inflammatory response and protecting the survival of the organism. Nevertheless, the complicated role of EcRIP2 in modulating cell survival and inflammation warrants further validation. Future investigations will need to deplete the genes of EcRIP2, EcASC, and EcCaspase-1 to identify the upstream signaling pathways and provide new ideas for virus disease control.

## Data availability statement

The datasets presented in this study can be found in online repositories. The names of the repository/repositories and accession number(s) can be found in the article/supplementary material.

## Ethics statement

The animal study was reviewed and approved by The Animal Care and Use Committee of South China Agricultural University.

## Author contributions

QQ and JW designed the experiments. XZ and SW performed the majority of the experiments and analyzed the data. XZ wrote the manuscript. ZL, HC, and JL contributed the experimental suggestions. All authors contributed to the article and approved the submitted version.
